# The endoplasmic reticulum stress response in aging and age-related diseases

**DOI:** 10.3389/fphys.2012.00263

**Published:** 2012-07-16

**Authors:** Marishka K. Brown, Nirinjini Naidoo

**Affiliations:** Center for Sleep and Circadian Neurobiology, University of PennsylvaniaPhiladelphia, PA, USA

**Keywords:** aging, age-related disease, UPR, BiP/GRP78, endoplasmic reticulum, stress

## Abstract

The endoplasmic reticulum(ER) is a multifunctional organelle within which protein folding, lipid biosynthesis, and calcium storage occurs. Perturbations such as energy or nutrient depletion, disturbances in calcium or redox status that disrupt ER homeostasis lead to the misfolding of proteins, ER stress and up-regulation of several signaling pathways coordinately called the unfolded protein response (UPR). The UPR is characterized by the induction of chaperones, degradation of misfolded proteins and attenuation of protein translation. The UPR plays a fundamental role in the maintenance of cellular homeostasis and thus is central to normal physiology. However, sustained unresolved ER stress leads to apoptosis. Aging linked declines in expression and activity of key ER molecular chaperones and folding enzymes compromise proper protein folding and the adaptive response of the UPR. One mechanism to explain age associated declines in cellular functions and age-related diseases is a progressive failure of chaperoning systems. In many of these diseases, proteins or fragments of proteins convert from their normally soluble forms to insoluble fibrils or plaques that accumulate in a variety of organs including the liver, brain or spleen. This group of diseases, which typically occur late in life includes Alzheimer's, Parkinson's, type II diabetes and a host of less well known but often equally serious conditions such as fatal familial insomnia. The UPR is implicated in many of these neurodegenerative and familial protein folding diseases as well as several cancers and a host of inflammatory diseases including diabetes, atherosclerosis, inflammatory bowel disease and arthritis. This review will discuss age-related changes in the ER stress response and the role of the UPR in age-related diseases.

## Introduction

Average life expectancies have been extended by as much as 30 years in developed countries during the Twentieth Century; a trend that is expected to continue in this century (Vaupel et al., 1998; Oeppen and Vaupel, 2002). The increase in elderly populations has raised interest in health consequences related to the aging process. A multitude of diseases that seemed rare many decades ago, are now amplified in aged individuals. Cases of dementia and Alzheimer's, incurable brain-wasting conditions, are expected to almost double every 20 years to around 66 million in 2030 and over 115 million in 2050 (Alzheimer's Association, 2012).

Evidence has implicated a role for unfolded/misfolded proteins in normal aging and age-related cognitive dysfunction. Age-associated deterioration of cellular machinery leads to an increase in the occurrence of protein misfolding, accumulation and aggregation, due in part to the gradual decay of chaperoning systems (Macario and Conway de Macario, 2002). In the majority of these diseases, proteins or protein fragments are transformed from their native soluble forms into insoluble fibrils or aggregated plaques that accumulate in a variety of organs. This group of conformational disorders, which includes Alzheimer's disease (AD), Parkinson's disease (PD), amyotrophic lateral sclerosis (ALS), Huntington's disease, type 2 diabetes mellitus, and a variety of other lesser known but equally severe conditions, appear later in life and are associated with aging. The fact that under normal physiological conditions, protein aggregates do not accumulate in the cells is partially due to the presence of cellular “quality control” mechanisms.

The endoplasmic reticulum (ER) contains one such system. The ER suppresses aggregation by accurately ensuring transcription and translation, chaperoning nascent or unfolded proteins, and discerning then transporting improperly folded polypeptides through a degradation pathway before they can aggregate (Ellgaard et al., 1999). Under conditions of stress, an adaptive mechanism that includes a set of coordinated signaling pathways termed the ER stress response or the unfolded protein response (UPR) is activated with the goal of returning the ER to its normal functioning state. In this review, we will examine key elements of the ER stress response, their age-related modifications, the effects of prolonged ER stress and the role of the ER stress response in several pathological disorders, many of which have implications for aging.

## Protein folding and quality control

In general, protein folding is a staggeringly inefficient process where some 30% of the proteins never acquire their fully folded conformation (Romisch, 2004). The ER is a membrane bound compartment and the ER lumen is topologically equivalent to the extracellular space. Its environment is highly oxidizing, which makes it suitable for protein folding and maturation. In mammalian cells, protein folding occurs in three phases (Naidoo, 2011). First, co-translational and co-translocational folding transpires as proteins traverse the ER membrane. After the release of the completed polypeptide from the ribosome, post-translational folding takes place.

Folding in the ER is the limiting step in the biogenesis of most secretory and transmembrane proteins. Chaperones and folding enzymes are engaged in all three folding stages. Key chaperones and folding sensors in the ER include: glucose regulated proteins 78 (GRP78; also known as Immunoglobulin Binding protein – BiP) and 94 (GRP94), the lectins, calnexin and calreticulin, and the thiol-disulfide oxidoreductases, protein disulfide isomerase (PDI) and ERp57. These components reside in the ER at high concentrations and participate in all stages of folding and in quality control.

Aging reduces the efficacy of many of these chaperones and foldases. Stringent restrictions on quality control within the ER prevent incompletely assembled or improperly folded proteins from exiting the ER and being transported to the cytosol or to downstream organelles and terminal compartments. Several abnormalities, including exposure of hydrophobic regions, unpaired cysteines and the tendency to form aggregates, will result in recognition of nascent proteins by the chaperones and folding sensors, which will cause their retention within the ER. Accumulation of misfolded proteins triggers ER stress and the UPR (Berridge, 2002).

## The ER stress response

**Figure 1:**
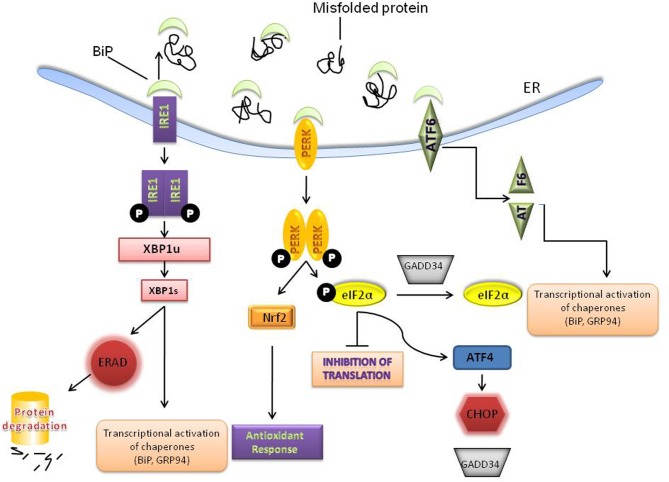
**Activation of the unfolded protein response (UPR).** Accumulation of misfolded or unfolded proteins in the ER leads to the dissociation of BiP from 3 transducers –PERK, IRE1 and ATF6. PERK homodimerizes and phosphorylates eIF2α to inhibit general protein translation. PERK also regulates several transcription factors including, NRF-2 to up-regulate the anti-oxidant response and ATF4 which can lead to both protective and apoptotic signaling. IRE-1 activation results in the unconventional splicing of XBP-1, which induces the transcription of several molecular chaperones, such as BiP and GRP94 and stimulates protein degradation via ER-associated degradation (ERAD). ATF6 is activated and cleaved and leads to induction of molecular chaperones. The various ER chaperones are part of a protective adaptive response that regulates protein folding and other components of the UPR.

Perturbations that alter ER homeostasis disrupt protein folding and lead to the accumulation of unfolded proteins and/ aggregates that trigger ER stress. ER stress can be provoked by a variety of physiological conditions, including perturbations in calcium homeostasis, glucose/energy deprivation, redox changes, ischemia, hyperhomocystinemia, and viral infections and mutations that impair client protein folding (Kaufman, 2002; Ron, 2002). As a consequence, the cell has evolved an adaptive coordinated response to limit the accumulation of unfolded proteins in the ER. These signaling pathways are collectively termed the ER stress response or the UPR [for more detailed reviews see (Zhang et al., 2001; Harding et al., 2002; Schroder and Kaufman, 2005; Walter and Ron, 2011)]. On a cellular level, the UPR triggers three kinds of protective cellular responses (see Figure 1 schematic): (i) up-regulation of ER chaperones such as BiP/GRP78 to assist in the refolding of proteins; (ii) attenuation of protein translation which is mediated by the serine-threonine kinase PERK which phosphorylates the initiation factor—eIF2α thereby reducing translation; and (iii) degradation of misfolded proteins by the proteasome by a process called ER associated degradation (ERAD). PERK activation also upregulates an antioxidant response through NF-E2-related factor 2 (Nrf2) (Johnson et al., 2008; Brown and Naidoo, 2010) (Figure 1). Nrf2 which belongs to the cap “n” collar (CNC) subfamily of basic leucine zipper transcription factors is known to play a significant role in the adaptive stress response to oxidative stress (Venugopal and Jaiswal, 1996; Itoh et al., 1999; He et al., 2001) and xenobiotic detoxification (Motohashi and Yamamoto, 2004). The three UPR responses are protective measures to limit protein load and alleviate ER stress; however, excessive and/prolonged stress leads to a maladaptive response and apoptosis (Szegezdi et al., 2006).

## The ER stress response signal is transduced by 3 proximal sensors

ER stress signals are transduced across the ER membrane by three proximal sensors of the UPR, inositol requiring element-1 (IRE-1), PKR like ER kinase (PERK) and activating transcription factor 6 (ATF6). All three of these sensors are maintained in an inactive state at the ER membrane by binding to the ER chaperone BiP (Immunoglobulin binding protein).

BiP is a peptide-dependent ATPase and member of the heat shock 70 protein family that binds transiently to newly synthesized proteins translocated into the ER, and more permanently to underglycosylated, misfolded, or unassembled proteins. BiP is also known as GRP78 and under the most recent nomenclature is known as HSP5A (Heat shock protein 5A). Under non-stress conditions BiP is bound to the three sensors ATF6, IRE1 and PERK, preventing them from activating downstream events. Upon accumulation of unfolded/ misfolded proteins, bound BiP dissociates from ATF6, IRE1 and PERK to chaperone the misfolded proteins thereby permitting the activation of one or more of these transducers (Zhang and Kaufman, 2006).

### PERK activation leads to the attenuation of protein translation

PERK is a type I transmembrane serine threonine kinase that appears to be present in most cells. It is held in an inactive monomeric state by binding to BiP. When this binding is disrupted, PERK homodimerizes and phosphorylates itself, becomes active and initiates its eIF2α kinase activity. Phosphorylation of the translation initiation factor, eIF2α, results in the formation of a stalled 43S ternary complex that causes a general decrease in translation of most proteins. However, some selected proteins with internal ribosomal entry sites (IRES), such as ATF4 and BiP, are translated more efficiently (Harding et al., 2000) and hence their protein levels actually increase.

### Activation of IRE-1 leads to splicing of XBP1

Once activated the cytoplasmic domain of IRE1α gains endoribonuclease activity and excises an intron from the mRNA encoding an UPR-specific transcription factor, X-box binding protein (XBP) 1, generating a spliced variant XBP1s. Spliced XBP1 functions as a potent transcriptional transactivator of genes involved in ER expansion, protein maturation, folding and export from the ER, as well as export and degradation of misfolded proteins (Yoshida et al., 2001; Calfon et al., 2002; Lee et al., 2002, 2003; Yoshida et al., 2003). ER-bound mRNAs are also degraded in an IRE1 dependent manner via a process called RIDD (“regulated IRE1-dependent decay”) and may serve to limit protein influx and unfolded protein load into the ER lumen after prolonged UPR induction (Hollien and Weissman, 2006; Pirot et al., 2007; Walter and Ron, 2011). Recent studies indicate an alternative model where IRE1 binds to unfolded proteins directly and that these serve as activating ligands (Walter and Ron, 2011).

### Activation of ATF6 increases the transcription of ER chaperones

ATF6 is a 90-kDa bZIP protein that is activated by posttranslational modifications. ATF6 activation as part of the UPR leads to its translocation to the Golgi and cleavage by site-1 protease (S1P) and S2P. The 50-kDa cleaved ATF6α translocates to the cell nucleus, where it binds to the ER stress response element CCAAT(N)9CCACG (Yoshida et al., 1998) in genes encoding ER chaperone proteins such as BiP and GRP94. GRP94 is a member of the heat shock90 family of chaperones. This binding results in increases in the level of these proteins and hence increased protein folding activity in the ER (Yoshida et al., 1998; Okada et al., 2002). Other important targets regulated by ATF6 include XBP-1, CHOP, HERP (hyperhomocysteinemia-induced ER stress responsive protein) and PDI (Protein disulfide isomerase).

## Sustained ER stress leads to an inflammatory response and apoptosis

**Figure 2:**
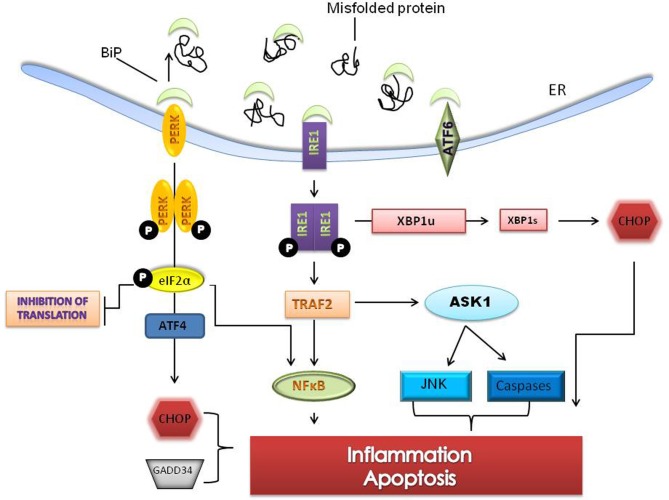
**Sustained ER stress leads to pro-apoptotic signaling and cell death.** Unresolved ER stress leads to inflammation and cell death pathways involving the PERK and IRE1 branches of the UPR. The IRE1-TRAF2- apoptosis signaling kinase 1 (ASK1) complex upregulates c-jun NH2 terminal kinase (JNK) and caspases and through splicing of XBP1 can activate C/EBP homologous protein (CHOP), a pro-apoptotic transcription factor. PERK signaling, through phosphorylation of eIF2α, can activate ATF4 dependent transcription resulting in activation of nuclear factor-kappaB (NFκB) and increases in CHOP. CHOP and ER specific caspases are thought to directly induce cell death. Several genes that mediate apoptotic function and inflammation are induced by prolonged ER stress.

Prolonged ER stress leads to inflammatory signaling while unmitigated and excessive stress leads to apoptosis (Szegezdi et al., 2006; Ron and Walter, 2007). Apoptosis in response to ER stress is specific to metazoan cells (Schroder and Kaufman, 2005). When cell protective changes mediated by the UPR fail to restore folding capacity, a combination of both the intrinsic and extrinsic apoptotic pathways are activated (Schroder and Kaufman, 2005). Apoptosis in response to ER stress is mediated largely by C/EBP homologous protein (CHOP) also known as growth arrest and DNA damage 153 (GADD 153). CHOP which is downstream of the PERK and ATF6 pathways induces the expression of a number of pro-apoptotic factors including Tribbles 3, GADD34 and DR5. Bcl-2 family members (Bak/Bax), caspase-12 and c-jun NH2 terminal kinase (JNK) are other components of the ER stress mediated apoptotic pathway (Wu and Kaufman, 2006) (Figure 2).

JNK activation, which occurs through IRE1 dependent signaling, induces the expression of inflammatory genes by phosphorylation of AP1 (transcription activator protein 1) (Davis, 2000; Zhang and Kaufman, 2008). NF-kappa B (NF-κB) dependent transcription is increased two ways during ER stress. First, I kappa K (IKK) which has a shorter half life is reduced when protein translation is attenuated thereby changing the stoichiometric ratio of NF-κB: IKK and freeing NF-κB to translocate to the nucleus (Zhang and Kaufman, 2008). Secondly, the IRE1-TRF2 complex recruits I kappa B (IKB) kinase that phosphorylates IKB and leads to its degradation (Hu et al., 2006).

## Age-related changes in the ER stress response

### UPR components decline with age

During aging, there is a shift in the balance between the protective adaptive response of the UPR and pro-apoptotic signaling; where the protective arm is significantly reduced and the apoptotic arm is more robust (Paz Gavilan et al., 2006; Hussain and Ramaiah, 2007; Naidoo et al., 2008). Key ER resident chaperones and enzymes within the ER such as BiP, PDI, calnexin and GRP94, which are required for proper protein folding, are impaired during the aging process. Chaperones are progressively oxidized with age and this process may contribute to their functional decline. These alterations in oxidation significantly correlate with reductions in enzymatic activity of several chaperones (Nuss et al., 2008).

BiP expression levels are significantly reduced in several species with age. In the cerebral cortex of aged (22–24-month old) C57/B6 mice compared with that in young (3-month old) mice, BiP protein levels were decreased 30% (Naidoo et al., 2008). BiP mRNA and protein expression levels are also decreased in the hippocampus of aged (23–26-month old) versus young (4–6-month old) Wistar rats (Paz Gavilan et al., 2006). A study that examined BiP protein expression in the brain (cortex and cerebellum) and in peripheral tissue (lung, liver, kidney, heart, and spleen) of Wistar rats across their life span found that expression of BiP was higher in the young tissue in comparison to the aged tissue (Hussain and Ramaiah, 2007).

Age modifies other components of the UPR in addition to the chaperones and enzymes. PERK mRNA was significantly reduced in the hippocampus of aged rats (Paz Gavilan et al., 2006). Another study reported that the activity of PKR (double stranded RNA-dependent kinase), an eIF2α kinase, was less efficient when isolated from aged rat brain tissue (Hussain and Ramaiah, 2007) than similar tissue isolated from young rats. Accompanying the declines in PERK signaling are increases in GADD34, which removes the translational block imposed by eIF2α phosphorylation. Increases in the expression of GADD34 were found in the cortical tissue of aged mice. Suppression of the translational block by GADD34 allows for the synthesis of pro-apoptotic proteins like CHOP.

Both basal and inducible CHOP expression levels are elevated with age (Kirkland et al., 2002; Ikeyama et al., 2003). Expression of CHOP and caspase-12, another pro-apoptotic molecule, was induced in aged rats that were stressed, but not in the young stressed animals (Paz Gavilan et al., 2006), lending support to the idea that the aged animals are more vulnerable to apoptosis. Studies from our laboratory demonstrate the upregulation of CHOP in aged mouse cortex (Naidoo et al., 2008) as others have shown in aged rat hippocampus (Paz Gavilan et al., 2006) and in aged rat cortex (Hussain and Ramaiah, 2007).

JNK kinases are also upregulated during aging. They are signal transduction proteins that regulate gene expression through the phosphorylation of transcription factors such as c-Jun and ATF-2. JNK is also activated by the kinase domain of IRE1 through the TNF receptor-associated factor 2 (TRAF2) and apoptosis signal-regulating kinase 1 (ASK1) (Ichijo et al., 1997; Szegezdi et al., 2006), which contributes to the induction of apoptosis.

### Structural changes

There are also structural changes in the ER with age. Hinds and McNelly, have demonstrated that the highly ordered parallel cisternae of rough ER characteristic of young neurons seems to become dispersed during aging (Hinds and McNelly, 2005). They have quantitatively measured this dispersion of the aging, rough ER cisternae in both mitral cells in the olfactory bulb and in Purkinje cells and found that there is a highly linear decrease in the measured closeness of rough ER cisternae throughout adult life. The age-related declines of important UPR chaperones, enzymes and ER structure significantly affect the efficiency of managing proper protein folding and ultimately ER homeostasis.

## Autophagy

The UPR activates autophagy in order to remove aggregates of misfolded proteins that cannot be degraded by the ERAD pathway (Ogata et al., 2006). Evidence suggests that autophagy can provide neuroprotection by enhancing clearance of these aggregates. Growing evidence indicates that autophagy also declines with age; the rate of autophagosome formation and maturation and the efficiency of autophagosome/lysosome fusion are reduced (Rajawat and Bossis, 2008). Dysregulation of the autophagic process may lead to neurodegeneration (Nedelsky et al., 2008). There are several excellent reviews that explore the relationship between ER stress, the UPR, autophagy and neurodegenerative disorders (Matus et al., 2008; Doyle et al., 2011).

## The ER stress response in age-related diseases

### Metabolic disorders and type 2 diabetes

The ER, which regulates protein synthesis and secretion as well as triglyceride and cholesterol biosynthesis and controls cellular metabolism, has been postulated to be a site for sensing metabolic stress. Recent data from experimental models indicate that ER stress is critical to the initiation and integration of pathways of inflammation and insulin action in metabolic syndrome (Hotamisligil, 2006). Metabolic syndrome which encompasses insulin resistance, reduced glucose utilization and type 2 diabetes are regulated by numerous mechanisms, including the UPR, JNK activation, NF-κB activation and apoptosis (Hotamisligil, 2006).

The two principal inflammatory pathways that disrupt insulin action, JNK–AP-1 and IKK–NF-κB, are linked to IRE-1 and PERK activity during ER stress (Deng et al., 2004). IRE-1 is linked to activation of JNK through a pathway involving TNF-receptor-associated factor 2 (Urano et al., 2000). Activation of both IRE-1 and PERK is also linked to the IKK–NF-κB pathway, through distinct mechanisms as described in the section above.

Insulin-receptor substrate 1 (IRS1), which transmits the effects of insulin through interactions with other cytosolic molecules is phosphorylated by activated insulin receptors on tyrosine residues. It is thought that JNK-mediated phosphorylation of serine residues in IRS1 inhibits the phosphorylation of IRS1 on tyrosine residues and leads to insulin resistance (Aguirre et al., 2002; Zhang and Kaufman, 2008).

### Sleep

It is becoming increasingly evident that sleep disruption leads to the ER stress response. Several studies have shown that acute sleep deprivation induces the up-regulation of BiP/GRP78 in the brains of mice (Naidoo et al., 2005; Mackiewicz et al., 2007), rats (Cirelli et al., 2004), birds (Jones et al., 2008) and fruitflies (Shaw et al., 2000; Naidoo et al., 2007). We have demonstrated that the PERK pathway is activated with six or more hours of sleep loss in the cerebral cortex of mouse brain (Naidoo et al., 2005). PERK cerebellar transcript levels were also found to be higher in wakefulness than in sleep (Cirelli et al., 2004). Other UPR specific transcripts that change with sleep deprivation include DNA-J which is a co-chaperone of BiP, XBP-1, calreticulin, caspase-9, ATF4 and ATF6.

Not only is the ER stress response activated with sleep loss it appears to be involved in the sleep homeostatic response. In Drosophila there is an increase in BiP with sleep loss and a diminution of expression with recovery sleep (Naidoo et al., 2007). BiP protein levels return to baseline levels over 24 h with recovery sleep following an almost 3-fold increase with 6 h of sleep deprivation. Over expression of BiP through genetic means leads to an increase in the amount of sleep recovered after sleep loss. Whether the altered amounts of recovery sleep when BiP levels are manipulated is due to BiP itself or more indirectly through other effects on the UPR remains to be determined.

#### Changes with age

Aged animals exhibit more fragmented sleep (Welsh et al., 1986; Shiromani et al., 2000; Naidoo et al., 2008) and display basal levels of ER stress in tissues examined (Naidoo et al., 2011). The adaptive ER stress response to sleep deprivation is impaired in aged mice cerebral cortices (Naidoo et al., 2008). There is little evidence of BiP up-regulation and attenuation of protein translation. Orexin and noradrenergic neurons in aged mice display considerable ER stress with activation of the PERK pathway when compared with similar regions in young mice (Naidoo et al., 2011). Surprisingly, recovery sleep following sleep deprivation is less in older animals than young. This has been shown in humans (Bonnet, 1985; Carskadon and Dement, 1987) and in rats (Mendelson and Bergmann, 2000; Shiromani et al., 2000). It is not known whether the UPR plays a role in the mammalian recovery sleep response to sleep deprivation and is currently being investigated.

### Neurodegenerative diseases

ER stress and activation of the UPR has been implicated in abnormal protein processing and neuronal death in age-associated diseases, which subsequently play a role in the pathogenesis of neurodegenerative diseases (see reviews Forman et al., 2003; Johnson et al., 2008). These diseases, which include Alzheimer's disease, Parkinson's disease, ALS and Huntington's disease, normally appear later in life and are thus associated with the aging process. Neuronal loss in both familial and sporadic forms of neurodegenerative disorders is often accompanied by aggregation of misfolded proteins (Selkoe, 2003). Studies have suggested that initial participation of the UPR in neurodegenerative disorders is probably cytoprotective, however, when activation of the UPR is sustained over an extended period of time, apoptotic pathways are upregulated. Accumulation of misfolded proteins that lead to alterations in organelle structure including the ER has been described in transgenic models of ALS, Alzheimer's and Huntington's disease (Reddy et al., 1999; Rao et al., 2002). Like many other signaling pathways, the UPR suffers from age-related impairments and becomes less effective (Naidoo et al., 2008).

Although many studies found activation of the UPR in neurodegenerative diseases, the studies are conflicting on whether there is induction of BiP. One study shows that BiP, along with other UPR markers were increased in the neurons of AD patients versus the non-demented controlled individuals (Hoozemans et al., 2005). Another study, on the other hand, found activation of the UPR, as measured by analyzing XBP-1 mRNA splicing along with activation of pro-apoptotic factors such as CHOP and caspases-3, 4, and 12; however, these authors show no induction of BiP expression (Lee et al., 2010). The conflict in each of these reports could be marked differences in the basal levels of BiP.

Lee et al. showed that UPR activation could be separated from amyloid β (Aβ) burden in the Tg2576 mice. Levels of PDI and BiP were similar in the cortex of aged Tg2576 and wild type mice (Lee et al., 2010). The authors suggest that Aβ burden alone is insufficient for induction of the UPR. This too is controversial, and rather ambiguous, since others have found that amyloid beta (Lee do et al., 2010; Costa et al., 2011), oligomeric not fibrillar, induces ER stress (Chafekar et al., 2007). A Drosophila model showed that neurotoxicity of tau was significantly increased when levels of XBP1 were reduced (Loewen and Feany, 2010). An earlier study, however, found that in an ALS murine model expressing mutant superoxide dismutase, removing XBP1 was neuroprotective, presumably through activation of an autophagic response (Hetz et al., 2009). P-PERK and P-IRE1 were found to be upregulated in response to early tau pathology (Nijholt et al., 2012).

Involvement of the UPR in Parkinson's disease (PD) has been described primarily in cellular models using drugs that mimic certain aspects of PD (Ryu et al., 2002; Smith et al., 2005). Parkin, an ubiquitin-protein ligase, is up-regulated in response to unfolded protein stress and suppresses cell death via its E3 activity. Loss of parkin function results in the misfolding and accumulation of PAEL-R, a substrate of parkin in the ER of substantia nigra neurons, leading to ER stress and cell death (Imai et al., 2001). This process has been proposed to be responsible for neuronal cell death in autosomal recessive juvenile parkinsonism and it suggests a physiological role of Parkin in dealing with ER stress (Imai et al., 2001). In addition, overexpression of wild-type or mutant α-synuclein induces UPR activation in yeast (Cooper et al., 2006). A study performed by Hoozemans and colleagues demonstrated activation of the PERK–eIF2a pathway in dopaminergic neurons in the substantia nigra of PD cases (Hoozemans et al., 2007). These varied studies suggest that targeting the UPR, whether by activation or inhibition, may provide opportunities for therapeutic intervention in several neurodegenerative disorders.

### Atherosclerosis and hyperhomocysteinemia

Evidence suggests that ER stress and the UPR can mediate the pathogenesis of atherosclerosis and vascular inflammation (Zhang and Kaufman, 2008; Santos et al., 2009). Atherosclerosis is a progressive disease of the large arteries where lipids and proteins derived from circulating low-density lipoproteins (LDL) accumulate within the cells and in the extracellular space (Ursini et al., 2002). There are a variety of methods in which ER function may influence the development of atherosclerosis. First, many critical lipid biosynthetic pathways are located in the ER (Gregor and Hotamisligil, 2007). Secondly, ER stress has been found to drive free-cholesterol-induced apoptosis in macrophages in a model of cellular free-cholesterol loading (Tabas, 2009). This suggests that the ER may sense stress related to lipid status and exposure, resulting in the passage of information to signaling pathways involved in inflammation and death in cells that are key in the development of atherosclerosis (Tabas, 2009). Finally, the UPR may have an important function in adaptive immunity. Substantial evidence indicates that autoimmune events mediated by ER stress may contribute to atherosclerosis (for a more detailed commentary on this subject see G. S. Hotamisligil, Nature Medicine, 2010) (Hotamisligil, 2010a,b).

Hyperhomocysteinemia (HHcy) is a common, independent risk factor for the development of cardiovascular disease. Epidemiological studies have found associations between high levels of serum homocysteine and the development of ischemic heart disease and stroke (Eikelboom et al., 1999); however, whether homocysteine is the underlying cause of atherosclerosis and thrombosis is not known. It was reported in cultured vascular endothelial cells that homocysteine induced protein misfolding in the ER by interfering with disulfide bond formation. This leads to activation of the UPR as the induction of several ER stress response proteins, such as BiP, GRP94, CHOP and HERP were found (Outinen et al., 1999; Huang et al., 2001). Homocysteine was also shown to activate apoptosis in an IRE1-dependent manner (Zhang et al., 2001). HHcy activates cleavage of the sterol regulatory element binding protein (SREBP), which leads to intracellular accumulation of cholesterol (Ron, 2001). Overexpression of BiP, which attenuates ER stress, suppresses this activation, implicating the UPR in the process (Kammoun et al., 2009; Basseri and Austin, 2012). The role of ER stress in atherosclerosis and other forms of cardiovascular disease likely involves an integrated network of multiple signaling pathways, including, but not limited to inflammation and lipid metabolism.

### The role of the UPR in cancer

Cancer rates increase sharply with age in both sexes, with the majority of cases occurring in patients over the age of 65 (Burkle et al., 2007). Recent studies in the cancer field have clearly demonstrated that ER stress and the UPR are robustly upregulated in various tumor types and are closely associated with cancer cell survival and their resistance to anti-cancer treatments (Wang et al., 2010). Researchers are now examining what markers of the UPR pathway are either induced or suppressed in malignancy in the hope of targeting these specific components for treatment. Tumor formation results in the high proliferation of cancer cells. During this process, a burden is placed on the ER that requires increased activities of protein folding, assembly and transport leading to physiological ER stress (Lee, 2007). As the tumor increases, the cancer cells are exposed to nutrient deprivation and hypoxic conditions; which are well known inducers for the accumulation and aggregation of unfolded and/or misfolded proteins in the ER, resulting in activation of the UPR pathways (Lee, 2007; Luo et al., 2009).

Evidence suggest that the adaptive arm of the UPR provides survival signaling pathways that are conducive to the growth of tumors, while suppressing the apoptotic arm of the UPR that would contribute to cell death and normal growth. Cancer cells may evade the apoptotic pathways by differentially activating the UPR branches (Pyrko et al., 2007; Hersey and Zhang, 2008). BiP has been shown to be upregulated in several different types of cancers (Zhang and Zhang, 2010) and is implicated in playing a critical cytoprotective role in oncogenesis (Li and Lee, 2006; Healy et al., 2009). BiP expression levels have been positively correlated with cerebral tumor malignancy, i.e., the higher the BiP levels, the more malignant the tumor (Zhang and Zhang, 2010). Previous reports found that BiP afforded protection against a variety of chemotherapeutic drugs that included: adriamycin, etoposide, 5-FU and temozolomide (Reddy et al., 2003; Fu et al., 2007; Lee, 2007; Pyrko et al., 2007). Later studies found that BiP was able to confer chemoresistance to tumor-associated endothelial cells (Virrey et al., 2008).

It has been reported by several groups that knocking down or interfering with BiP function, sensitizes tumors to treatment. Wang et al found that (-)-epigallocatechin gallate (EGCG), a natural inhibitor of BiP that targets its ATP-binding domain, sensitizes breast cancer cells to taxol and vinblastine, two broad cytotoxic drugs (Wang et al., 2009). Another study found that down-regulation of BiP by small interfering RNA decelerates glioma cell growth (Pyrko et al., 2007).

Other components of the UPR are also activated in cancer. When the spliced variant of XBP1 was expressed in IRE1α dominant-negative expressing cells angiogenesis was restored (Romero-Ramirez et al., 2009), suggesting that signaling through the IRE1α-XBP1 arm of the UPR is essential for angiogenesis in the early stage of tumor development.

The PERK-eIF2α branch of the UPR has been shown to protect cells under conditions of hypoxia; a feature common to solid tumors that is the result of increased demands on energy requirements due to dysregulated cell growth (Wouters and Koritzinsky, 2008). Hypoxic conditions can induce PERK, leading to phosphorylation of eIF2α in tumor cells (Koumenis et al., 2002; Fels and Koumenis, 2006). Evidence supporting the role of the UPR in cancer offers some interesting therapeutic alternatives for cancer treatment. Approaches to induce and/or prevent UPR activation in oncogenesis could have implications for improving therapeutic outcomes.

### Other age-related inflammatory diseases

Besides the well known age–related diseases and disorders discussed above, ER stress has been implicated in several chronic diseases involving inflammation (Hotamisligil, 2010a,b). These include neuromuscular inflammatory diseases, arthritis and spondyloarthropathies, multiple forms of respiratory inflammation and inflammatory bowel diseases (Hybiske et al., 2007; Mhaille et al., 2008; Colbert et al., 2010; McGuckin et al., 2010). In many of these diseases, it is not yet clear whether ER stress is a primary contributor to the disease or a consequence of the condition. It has been suggested that, in some circumstances, ER stress can initiate disease but that inflammation, in some cases owing to infection, is an important exacerbator of ER stress and can be the trigger for the onset of disease in a genetically susceptible individual [for more information see review by (Hasnain et al., 2012)]. Increasing evidence links ER stress to inflammatory bowel disease. Secretory epithelial cells that produce anti-microbial molecules and the mucus barrier, which separate the epithelium from the luminal microbes, are very vulnerable to ER stress (Hasnain et al., 2012). These cells produce the high molecular weight cysteine rich mucins that are prone to misfolding. Genetic studies in rodent models indicate that deficiencies in the UPR pathway lead to spontaneous intestinal inflammation (Heazlewood et al., 2008).

## Concluding remarks

The ER stress response comprises a complex set of signaling pathways that serves to maintain ER and protein homeostasis in response to genetic, host (hypoxia, ATP, calcium alterations), microbial and inflammatory stressors. With age, many of the components of the UPR decline and become less efficient thus dampening the response to these stressors. This results in or exacerbates existing diseases. Therapeutics that reduce ER stress by promoting correct folding of proteins, improving the efficiency of ERAD and/or enhancing the detection of misfolded proteins may prove useful in delaying or preventing some of the age-related diseases and disorders discussed in this review. Also, recognizing perturbations in the ER stress response could lead to early detection of a number of age-related pathologies and the development of therapeutics which maintain a normal ER stress response later in life. This represents a hitherto unexplored avenue to disease prevention.

### Conflict of interest statement

The authors declare that the research was conducted in the absence of any commercial or financial relationships that could be construed as a potential conflict of interest.
